# Glycolysis in Panc-1 human pancreatic cancer cells is inhibited by everolimus

**DOI:** 10.3892/etm.2012.787

**Published:** 2012-11-01

**Authors:** LING LIU, LIANSHENG GONG, YANGDE ZHANG, NIANFENG LI

**Affiliations:** National Hepatobiliary and Enteric Surgery Research Center, Xiangya Hospital, Central South University, Changsha, Hunan 410008, P.R. China

**Keywords:** everolimus, pancreatic cancer, Panc-1 cells, apoptosis, glycolysis

## Abstract

The aim of this study was to evaluate the effects and molecular mechanisms of everolimus on Panc-1 human pancreatic cancer cells. Panc-1 human pancreatic cancer cells were treated with everolimus (10 μg/ml) at selected time points (6, 12 and 24 h). Cell proliferation and apoptosis were evaluated by MTT and flow cytometric analyses. The glycolytic activity was determined by measuring the activity of the key enzyme lactate dehydrogenase (LDH) and lactate production. The activity of mammalian target of rapamycin (mTOR) signaling was measured by western blotting. The expression of genes, including hexokinase 2 (HK2) and microRNA-143 (miR-143), was evaluated by real-time polymerase chain reaction (PCR). The administration of everolimus time-dependently inhibited proliferation and glycolysis and induced apoptosis in the Panc-1 human pancreatic cancer cells. As the time of treatment with everolimus increased, the mTOR signaling activity decreased, indicated by lower phosphorylation levels of S6 kinase; however, the phosphorylation levels of mTOR barely changed. Moreover, our data showed an everolimus-induced increase in miR-143 and decrease in HK2 in Panc-1 cells in a time-dependent manner. In conclusion, the current study indicates a novel role of everolimus in its antitumor effect as an inhibitor of glycolysis in Panc-1 human pancreatic cancer cells. Furthermore, our data highlights the significance of exploring the mechanisms of everolimus and miR-143 in malignant tumors.

## Introduction

Pancreatic cancer, with a 5-year survival rate of 5%, is one of the most common gastrointestinal malignant tumors with an exceptionally poor prognosis. Even among those patients who undergo resection and have tumor-free margins, the 5-year survival rate is only 10 to 25% (1). Once pancreatic cancer becomes metastatic, it is uniformly fatal with an overall survival of typically 6 months from diagnosis. Over the past 30 years, although combined strategies including surgery, radiotherapy and chemotherapy, have been gradually applied to the treatment of pancreatic cancer, there has been no significant improvement in survival rate (2). Therefore, an effective therapeutic strategy is urgently needed.

Over 70 years ago, Warburg observed that cancer cells exhibit enhanced conversion of glucose to lactate (aerobic glycolysis) and preferentially metabolize glucose through glycolysis to meet their energy needs, even in the presence of an adequate level of oxygen (3). This phenomenon was then named the Warburg effect. In solid tumors, the generation of neovasculature always lags behind the rapid expansion of the tumor mass, resulting in the lack of oxygen delivery and hence local ischemia and hypoxia (4). The Warburg effect demonstrates that cancer cells are less dependent on oxygen and satisfy themselves with adequate energy and molecules including ATP, nucleotides and fatty acids, necessary for the rapid growth of cancer cells. During the past decades, the Warburg effect has been identified in multiple types of human malignant tumors, including pancreatic cancer (5). Thus, the development of therapeutic agents targeting the Warburg effect may provide an effective strategy for the treatment of pancreatic cancer.

Chemotherapy has been demonstrated to be useful for improving quality of life and gaining a modest survival benefit for patients with pancreatic cancers. At present, novel chemotherapeutic drugs are needed for the treatment of patients with pancreatic cancer (6). Everolimus is an analog of rapamycin, which is a natural compound that has a potential antitumor property (7). The anti-proliferative effect of everolimus makes it a promising therapeutic agent for inhibiting tumor growth. In 2011, everolimus was approved by the Food and Drug Administration (FDA) for use against pancreatic neuroendocrine tumors (8). Therefore, understanding the molecular mechanisms of everolimus’ antitumor effects may help in the development of strategies for the therapy of pancreatic cancer. However, whether everolimus influences microRNAs and glycolysis in pancreatic cancer remains unclear.

In the current study, everolimus showed time-dependent anti-proliferative and pro-apoptotic effects in Panc-1 human pancreatic cancer cells, possibly by inhibiting mTOR signaling. Moreover, everolimus inhibited cellular glycolysis in Panc-1 cells in a time-dependent manner, which may partly be explained by the upregulation of miR-143 and the deregulation of HK2, the latter of which plays an essential role in glycolysis in cancer cells.

## Materials and methods

### Cell line and cell culture

The human pancreatic cancer cell line Panc-1 was used in this study. Panc-1 cells were routinely cultured in Dulbecco’s modified Eagle’s medium (DMEM) containing 10% fetal bovine serum (FBS) at 37°C with 5% CO_2_. For experiments, cells were cultured either with or without FBS stimulation. Everolimus was dissolved in dimethylsulfoxide (DMSO) and used in cell culture at a final concentration of 10 μg/ml. The Panc-1 cells in experimental groups were treated with everolimus for 6, 12 or 24 h.

### MTT assays

Cells were plated at a density of 5,000 cells per well and then treated with 2% (v/v) DMSO (control) or with everolimus (10 μg/ml) for 6, 12 and 24 h. MTT (Promega, Madison, WI, USA) was added to the medium at a final concentration of 0.5 μg/ml. Cells were then incubated at 37°C with 5% CO_2_ for 3 h. The medium was removed and 100 μl DMSO was added to each well. The plate was gently rotated on an orbital shaker for 10 min to completely dissolve the precipitate. The absorbance was detected at 570 nm with a microplate reader (Bio-Rad, Hercules, CA, USA).

### Flow cytometric analyses

Panc-1 cells (1×10^5^) were seeded in 6-well plates and treated with everolimus for 6, 12 or 24 h. Cells were then collected and washed twice with phosphate-buffered saline (PBS) and resuspended in 400 μl binding buffer. Annexin V-fluorescein isothiocyanate (FITC) and propidium iodide (PI) staining (5 μl Annexin V-FITC and PI solution) were used to visualize apoptotic cells according to the manufacturer’s instructions. Samples were incubated for 15 min at room temperature and analyzed using a flow cytometer (Beckman Coulter, Miami, FL, USA).

### Measurement of glycolytic activity

The cellular glycolytic activity was determined by measuring the activity of the key enzyme lactate dehydrogenase (LDH) and lactate production. Since LDH catalyzes the reaction: L-lactate + nicotinamide adenine dinucleotide (NAD)^+^ = pyruvate + NADH, the activity of LDH was measured spectrophotometrically by the increase in NADH at 340 nm. The lactate concentration in the culture medium was measured using using the lactate assay kit (BioVision, Mountain View, CA, USA).

### Western blotting

The Panc-1 cells were solubilized in cold radioimmunoprecipitation assay (RIPA) lysis buffer and then separated with 5% sodium dodecyl sulfate-polyacrylamide gel electrophoresis (SDS-PAGE). Following SDS-PAGE, the proteins were transferred to a polyvinylidene fluoride (PVDF) membrane. Membranes were blocked in 5% non-fat dried milk in PBS for 3 h and then incubated overnight with specific antibodies for p-mTOR, p-S6 kinase and tubulin (Abcam, Cambridge, UK). Following incubation with the second antibody (Abcam), immune complexes were detected using the enhanced chemiluminescence (ECL) method. Results were visualized by autoradiography using preflashed Kodak XAR film (Kodak, Tokyo, Japan).

### Real-time reverse transcription-polymerase chain reaction (RT-PCR)

Total RNA was extracted from cultured cells using the RNeasy RNA isolation kit (Qiagen, Hilden, Germany) and then reverse transcribed into cDNA using the miScript II RT kit (Qiagen). cDNA was then amplified by real-time PCR using SYBR-Green dye Universal Master mix on a LightCycler 480 instrument with the miScript primer of the HK2 gene for 40 cycles. Glyceraldehyde 3-phosphate dehydrogenase (GAPDH) served as a housekeeping normalization control. The relative amount of mRNA compared with the GAPDH level was calculated using crossing point (Cp) values and scaled relative to control samples set at a value of 1. Results for gene expression in experimental samples were plotted compared with the control. Mature miR-143 levels were quantified using the TaqMan^®^ microRNA assay (Applied Biosystems, Carlsbad, CA, USA) and normalized to the U6 small nuclear B non-coding RNA (Applied Biosystems).

### Statistical analysis

Statistical analysis was performed using SPSS 15.0 statistical software (SPSS Inc., Chicago, IL, USA). Data were expressed as the mean ± standard deviation (SD) of triplicate experiments and analyzed by one-way analysis of variance (ANOVA) or t-test for multiple comparisons. P<0.05 was considered to indicate a statistically significant difference.

## Results

### Effect of everolimus on the proliferation of Panc-1 cells

**Figure 1:**
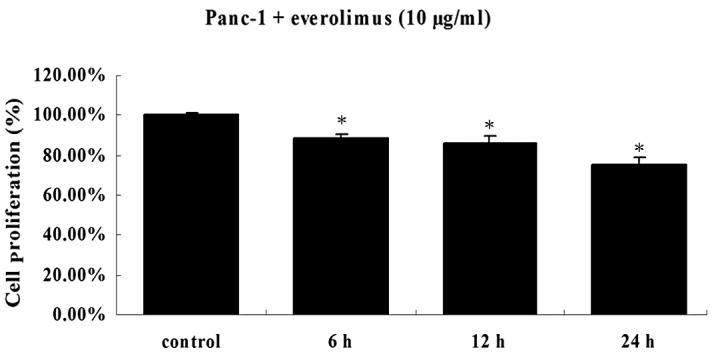
Everolimus inhibits proliferation of Panc-1 cells. Cells were plated at a density of 5,000 cells per well and then treated with 2% (v/v) DMSO (control) or with everolimus (10 μg/ml) for 6, 12 and 24 h. Cell viability was then determined by an MTT assay. ^*^P<0.05, compared with the control. DMSO, dimethylsulfoxide.

In the current study, we tested the *in vitro* anti-proliferative effect of everolimus on the pancreatic cancer cell line Panc-1. As shown in Fig. 1, following everolimus treatment for 6, 12 and 24 h, the average cell proliferation rate was reduced to 88.30, 85.93 and 75.32% of the control rate, respectively. Therefore, these results suggested that everolimus inhibited the proliferation of Panc-1 cells in a time-dependent manner.

### Effect of everolimus on apoptosis in Panc-1 cells

**Figure 2:**
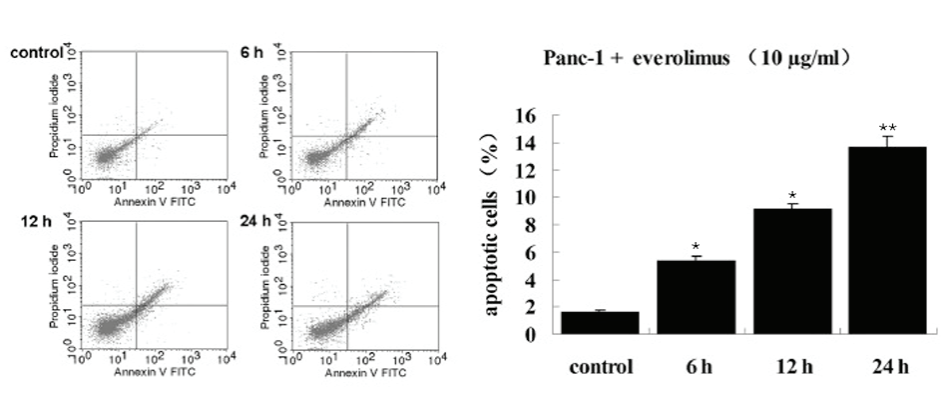
Everolimus induced cell apoptosis in Panc-1 pancreatic cancer cells. Following treatment of Panc-1 cells with 10 μg/ml everolimus for 6, 12 and 24 h, apoptotic cells were detected by Annexin V and PI double staining. Panc-1 cells which were not treated with everolimus were used as a control. (^*^P<0.05, ^**^P<0.01, compared with the control). PI, propidium iodide; FITC, fluorescein isothiocyanate.

We then analyzed the percentage of apoptotic cells in the control group and each experimental group, in order to determine whether the anti-proliferative effect of everolimus was accompanied by induced apoptosis. It is well known that the disturbed balance between cell proliferation and cell death plays an essential role in the development of cancers. In Panc-1 cells, following everolimus treatment for 6, 12 and 24 h, the average percentages of apoptotic cells were 5.35, 9.17 and 13.72%, respectively, while the average percentage of apoptotic cells in the control culture was only 1.65% (Fig. 2). These results revealed that everolimus enhanced the apoptosis of Panc-1 cells in a time-dependent manner.

### Effect of everolimus on glycolysis in Panc-1 cells

**Figure 3:**
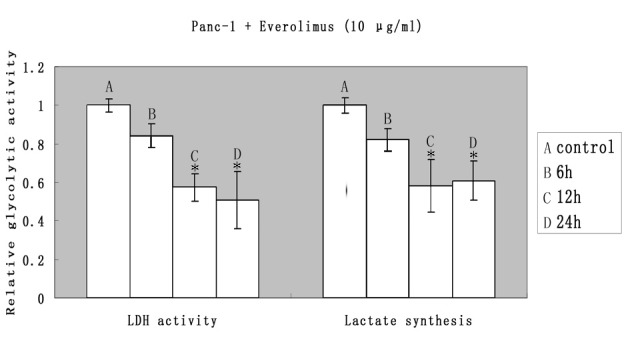
Everolimus inhibited glycolysis in Panc-1 pancreatic cancer cells. Panc-1 human pancreatic cancer cells were treated with everolimus (10 μg/ml) for 6, 12 and 24 h. Panc-1 cells that were not treated with everolimus were used as a control. The LDH activity and lactate synthesis in each group were measured as described in Materials and methods. The levels of LDH activity and lactate synthesis decreased significantly following treatment with everolimus for 12 and 24 h. ^*^P<0.05, compared with the control. LDH, lactate dehydrogenase.

In order to verify that everolimus inhibits glycolysis in Panc-1 cells, we measured the activity of LDH, a key enzyme involved in glycolysis, as well as lactate production in each experimental and control group. As illustrated in Fig. 3, Panc-1 cells treated with everolimus demonstrated significantly lower levels of LDH activity and lactate production, compared with Panc-1 cells that were not treated with everolimus.

### Everolimus suppressed the phosphorylation of S6 kinase

**Figure 4:**
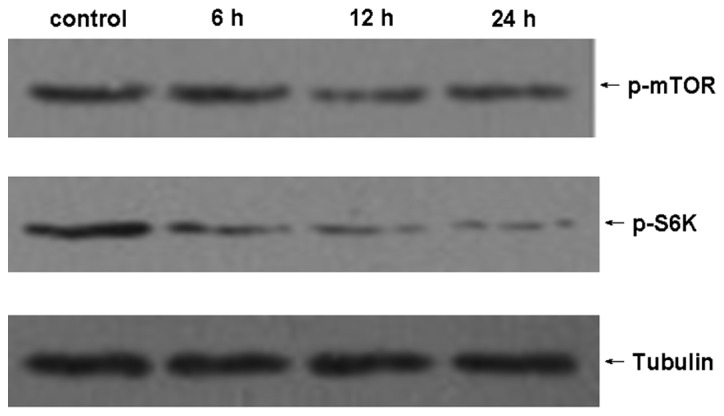
Everolimus suppressed the protein expression of mTOR. In the experimental groups, Panc-1 cells were treated with everolimus (10 μg/ml) for 6, 12 and 24 h. Panc-1 cells which were not treated with everolimus were used as a control. Each group of Panc-1 cells was lysed and western blotting was performed with p-S6 kinase and p-mTOR antibodies. Immunoblotting by anti-tubulin was performed for the control. mTOR, mammalian target of rapamycin.

To investigate the molecular mechanism of everolimus and its antitumor effect in Panc-1 cells, we further examined the effects of everolimus on the phosphorylation levels of mTOR and S6 kinase, a direct substrate of mTOR. As shown in Fig. 4, during the prolonged treatment with everolimus, although the phosphorylation level of mTOR in Panc-1 cells barely changed, the phosphorylation level of S6 kinase was gradually deregulated in a time-dependent manner. These results indicate that everolimus time-dependently inhibits mTOR signaling in Panc-1 cells.

### Everolimus upregulated miR-143 and downregulated HK2 in Panc-1 cells

**Figure 5:**
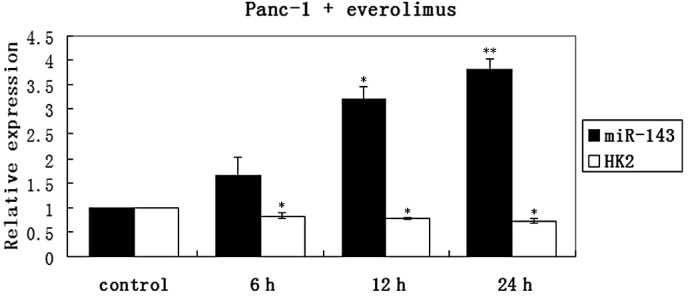
Everolimus upregulated miR-143 and downregulated HK2 in Panc-1 cells. After treatment with everolimus (10 μg/ml) for 6, 12 or 24 h, the relative expression of miR-143 and its target gene HK2 in Panc-1 cells were detected using real-time RT-PCR. The histogram plots demonstrate the relative expression levels of miR-143 and HK2 in each group. The levels of miR-143 and HK2 changed significantly following treatment with everolimus for 12 and 24 h. ^*^P<0.05, ^**^P<0.01, compared with the control. HK2, hexokinase 2; RT-PCR, reverse transcription-polymerase chain reaction.

It has been reported that miR-143 is regulated by mTOR and that HK2, a key enzyme involved in glycolysis, is a direct target of miR-143. To investigate whether miR-143 and HK2 were affected by the administration of everolimus, we applied real-time RT-PCR to measure miR-143 and HK2 levels in Panc-1 cells treated with everolimus for varying times. Panc-1 cells that were not treated with everolimus were used as a control. As shown in Fig. 5, during the treatment of Panc-1 cells with everolimus, the upregulation of miR-143 was time-dependently accompanied by the downregulation of HK2.

## Discussion

Everolimus has been suggested to regulate various cellular biological behaviors, including the inhibition of proliferation and the induction of apoptosis in multiple types of malignant tumors (9–12). In the present study, we showed that as the treatment of the Panc-1 cells with everolimus was prolonged, cell proliferation gradually decreased, while the apoptotic rate gradually increased. Therefore, our findings indicate that everolimus has the potential to be used as a promising therapeutic agent for the therapy of pancreatic cancer.

mTOR signaling is abnormally upregulated in multiple types of cancers and has been explored as a potential therapeutic target in patients with pancreatic cancer (13). mTOR is the target of rapamycin, acting as a major downstream signaling molecule of phosphatidylinositol 3′-kinase (PI3K)/Akt. It has been universally accepted that mTOR has a great influence on multiple cellular functions, including cell survival, proliferation and differentiation; this is mainly attributed to two different complexes, mTOR complex 1 (mTORC1) and complex 2 (mTORC2) (14). mTORC1, composed of mTOR, raptor and mLST8, enhances the translation of many mRNAs through phosphorylation of S6 kinase, which induces cell growth (15). mTORC1 is regulated by various molecules, including rapamycin and its analogs. Everolimus, as a rapamycin analog, is an inhibitor of mTORC1 (16). Several studies have demonstrated that everolimus inhibits the growth of several cancer cell lines *in vitro* and inhibits tumor growth in animal models (12,17). However, the detailed mechanism of everolimus in the treatment of pancreatic cancer remains unclear. Data in the present study revealed that during the treatment of Panc-1 cells with everolimus, mTOR signaling decreased in a time-dependent manner, indicated by the decreased phosphorylation of S6 kinase. Given that mTOR has an influence on cell survival and apoptosis, our data suggests that decreased mTOR signaling is likely to be associated with the everolimus-induced anti-proliferative effects and apoptosis observed in the Panc-1 cells.

The Warburg effect is observed as a promising target of therapy for cancers. In the current study, the glycolytic activity was significantly inhibited by treatment with everolimus. We further explored the molecular mechanism. It has been suggested that the main cause of the Warburg effect in cancer cells is the abnormal upregulation of key enzymes, including HK2, the most important enzyme involved in the first step of glycolysis (18). The abnormal overexpression of HK2 may lead to the shift towards aerobic glycolysis (19). In the present study, everolimus effectively inhibited the mRNA level of HK2, which partly explains the decreased level of glycolysis in the Panc-1 cells. A previous study reported that HK2 is a direct target of miR-143 and may be downregulated by it (20). miR-143 has been shown to be downregulated in various types of human cancers (21–33) and forced expression of miR-143 effectively inhibits the growth, invasion and migration of cancers (34,35). Futhermore, the miR-143-mediated HK2 regulation may also exist in breast and colon cancer (36,37), suggesting a common mechanism of miR-143 repressing HK2. Therefore, we further analyzed the expression of miR-143 and verified that with the prolonged treatment with everolimus, the miR-143 level was gradually upregulated. Additionally, in lung adenocarcinoma cell lines, the expression of miR-143 was found to be repressed by mTOR (20) and the mTOR signaling was inhibited by everolimus in our study. Collectively, for the first time we have demonstrated that everolimus inhibits glycolysis, partly by repressing mTOR signaling which then upregulates miR-143, an important cancer-related microRNA involved in the deregulation of HK2.

In conclusion, the current study highlights a new antitumor mechanism of everolimus, which is a promising therapeutic agent for human pancreatic cancer. With the data from the current and other studies, we have demonstrated that, besides the inhibition of cell proliferation and the induction of cell apoptosis by downregulating the mTOR signaling, everolimus also inhibits glycolysis in Panc-1 cells, partly by the upregulation of miR-143 and the deregulation of HK2. In addition, the present study has also helped to better explain the functions of miRNAs in cancer cells. With further follow-up studies, research on the detailed molecular mechanisms of everolimus should shed light on the treatment of pancreatic cancer.
